# Association of shared decision-making cessation model and adult smoking cessation rate: A prospective cohort study

**DOI:** 10.18332/tid/200023

**Published:** 2025-01-31

**Authors:** Kuan-Lun Chen, Yun-Chen Hsu, Yi-Hsuan Li, Fei-Ran Guo, Jaw-Shiun Tsai, Shao-Yi Cheng, Hsien-Liang Huang

**Affiliations:** 1 Department of Medical Education, National Taiwan University Hospital, Taipei, Taiwan, China; 2 Department of Family Medicine, National Taiwan University Hospital, Taipei, Taiwan, China; 3 Department of Nursing, National Taiwan University Hospital, Taipei, Taiwan, China

**Keywords:** medication adherence, shared decision making, smoking cessation

## Abstract

**INTRODUCTION:**

It remains unclear whether shared decision-making (SDM) can help smoking cessation. This study aims to determine whether the SDM model increases the 24-week point abstinence rate and medication adherence rate for adult smokers.

**METHODS:**

This prospective cohort study, conducted between January 2019 and June 2021, enrolled 1268 adult smokers at the outpatient cessation clinic of a national medical center. SDM-integrated counseling was provided to those opting for the SDM cessation model, involving cessation educators and decision aids. Patients who declined the model received cessation medication. The self-reported 7-day point prevalence abstinence rate at week 24, medication adherence rate, and the proportion of participants agreeing to receive pharmacotherapy were measured.

**RESULTS:**

Out of the 1268 participants, 1187 (93.6%) were included in the primary analysis. Of these, 610 (48%) opted for the SDM model. Participants in the SDM group used cessation medication more frequently (83.4% vs 71.9%, p<0.001) and exhibited higher medication adherence (39.1% vs 28.6%, p=0.04). Logistic regression analysis revealed that the SDM group did not demonstrate a significantly higher 7-day point abstinence rate at week 24 (OR=0.89; 95% CI: 0.68–1.15; p=0.37).

**CONCLUSIONS:**

The SDM cessation model was positively associated with medication adherence and the proportion of participants using pharmacotherapies. However, the association of SDM with the 7-day point prevalence of abstinence at week 24 was not statistically significant. Longer follow-up studies are needed to understand the association of the SDM intervention with absolute abstinence.

## INTRODUCTION

Smoking is a serious public health concern. An estimated 1.27 billion people are expected to smoke in 2025 due to population growth^1^. Tobacco-related diseases including malignant neoplasms, cardiovascular diseases, and respiratory diseases, account for over 8 million deaths annually^1-6^.

Common cessation methods are a combination of pharmacotherapies, behavioral support, and motivational support^7^. By increasing the medication adherence rate, the 6-month cessation rate could be doubled^8^. Behavioral counseling and pharmacotherapy improve the cessation rate among the general adult population, increasing cessation by 82% compared with minimal intervention or usual care^9^. Nicotine replacement therapy and non-nicotine pharmacotherapy result in approximately a 20% cessation rate with a possible ceiling effect according to previous research^10-12^. Limitations regarding current methods include insufficient compliance and adherence to medication^13,14^. Factors affecting adherence include experience, motivation, confidence, nicotine dependence, and patients’ perceptions, beliefs, and knowledge about treatments^14,15^.

Shared decision-making (SDM) is a collaborative approach improving the quality of decisions by reducing conflict and shifting the power and control of interactions between patients and physicians, while highlighting patients’ autonomy^16^. Similar ideas in smoking cessation are behavioral counseling and motivational interviewing, with evidence of increasing cessation rates^17-19^. A systematic review indicated that SDM has a positive association with treatment adherence and satisfaction with the treatment of chronic illness^20^. A review also showed that participant-centered adherence interventions that consider perceptions towards medication increased cessation rates^21^. According to a previous study, decision aids might be helpful in increasing the knowledge of smoking cessation methods, decisional quality, and quit attempts^22^. Furthermore, the decision aid was designed to help the process of SDM by offering clear information, including comparisons between different cessation methods. However, there are currently insufficient studies exploring the association between using the SDM model in cessation clinics and smoking cessation rates or medication adherence.

Previous studies have shown that expert advice fosters the transition between the five transtheoretical model (TTM) stages and that TTM-based stage-matched intervention, though controversial, might have a positive effect on the cessation rate^23,24^. Thus, the proposed model focused on reinforcing the transition between the preparation and action stages of TTM with the help of SDM, thereby achieving a higher cessation rate.

This prospective cohort study aimed to determine the association among SDM, abstinence, and treatment adherence rates for tobacco cessation. This study hypothesized that SDM could maximize the efficacy of pharmacotherapy by increasing treatment adherence rate.

## METHODS

### Study design and study sample

**Figure 1:**
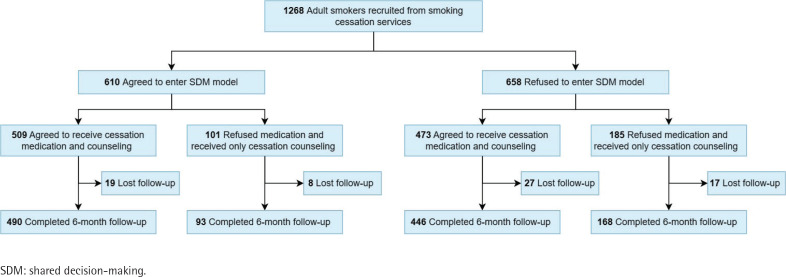
Participant flow diagram showing number of participants recruited, grouped, followed-up, and analyzed, Taipei, Taiwan, January 2019 to June 2021 (N=1268)

The study was designed as a prospective cohort study conducted between January 2019 and June 2021 at a national medical center in Taipei, Taiwan. The participants were recruited from cessation clinic patients. All participants provided informed consent to participate. The participant flow is shown in Figure 1. If the adult smokers recruited from the smoking cessation services agreed to enter the SDM model, an SDM counseling session between a specialized cessation educator and a patient decision aid (PDA) was provided before counseling and medication. Participants could opt to receive counseling alone or in combination with medication. All participants were followed up for 6 months. Patients visiting the smoking cessation clinic were eligible to enroll if they were aged >20 years and were able to understand the contents of PDA. Patients who could not understand the contents of PDA were excluded. This study was approved by the Institutional Review Board of the National Taiwan University Hospital (201806018RIND).

### Framework of the SDM model for smoking cessation

**Figure 2:**
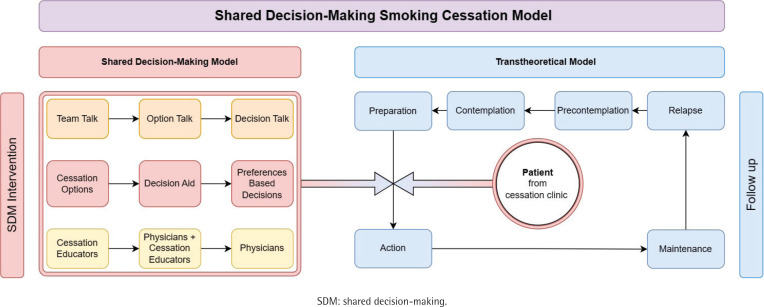
Framework of shared decision-making cessation model applied to the SDM group

Participants in the SDM group received an SDM session based on the model shown in Figure 2. The SDM model was designed based on the three-talk model of SDM to help participants effectively transition from the preparation stage to the action stage of TTM^16^. Participants were in the preparation stage when recruited from the cessation clinic. The goal of the SDM session is to foster and strengthen the transition from preparation to the action stage according to patient informed preference.

The SDM model consists of the following three main steps: ‘team talk’, ‘option talk’, and ‘choice talk’. Through the process of SDM, the autonomy of people who smoke is highlighted, and the rapport to work together with the cessation educators for cessation is established in the ‘team talk’ stage. ‘Option talk’ offers a detailed comparison between the available cessation medications, including the absolute cessation rate, benefits, costs, and potential adverse effects of each medication. By comparing the risks and benefits of the treatment options, the patient will have sufficient knowledge to make choices. People who smoke would also be better prepared to face possible adverse events. Afterwards, a joint decision based on the informed preferences of participants is made in the ‘decision talk’. Participants’ involvement in the decision process is ensured during the SDM process.

*Decision aid*

The decision aid was a structured pamphlet with three parts: 1) an overview of the pros and cons of different smoking cessation managements including cessation counseling only, cessation counseling with nicotine replacement therapy, and cessation counseling with non-nicotine replacement therapy; 2) questions that help adults who smoke to clarify their preferences; and 3) the SURE (Sure of myself, Understand information, Risk-benefit ratio, Encouragement) test for SDM quality assessments. The detailed information on the first part of the decision aid included fees, advantages, limitations, usage, and adverse effects, intending to minimize information asymmetry and reassure patients about optimizing their decisions. With the SURE test reassuring the decision made by the participants, decisional conflict could be avoided, thereby finding the most suitable choice of cessation method based on their own preferences instead of physicians’ suggestions alone^25^. The process of the decision support tool development can be found in previous research^26^.

*Pharmacotherapy*

The cessation medications for the subgroups of participants who agreed to use medication treatment in both the SDM and non-SDM groups included nicotine replacement therapies (nicotine patch and gum) and non-nicotine pharmacotherapy (varenicline). Participants within the SDM group chose the option of therapy after the SDM counseling session as a joint decision, while those in the non-SDM group received the most suitable medications chosen by physicians specialized in smoking cessation. Medications were prescribed at intervals of 1, 1, 2, 4 weeks or 2, 2, 4 weeks as patients’ preferences, and were fully subsidized by the Health Promotion Administration, Ministry of Health and Welfare, Taiwan. The total amount of medication subsidized was 16 weeks a year.

*Follow-up cessation counseling*

All participants received 10–15 min of cessation counseling in the form of standardized motivational interviews during the follow-up visits provided by physicians specialized in smoking cessation and cessation educators. Baseline assessments were performed during the first visit to the cessation clinic. During each consultation at the return visit, at intervals of 1, 1, 2, 4 weeks or 2, 2, 4 weeks, the participants were reassessed by physicians and cessation educators. Baseline assessments included demographics, smoking status, and Fagerström test for nicotine dependence (FTND). The follow-up assessments included smoking status and medication adherence. The condition of medication usage, including adherence and adverse effects, was assessed at every follow-up cessation counseling session by the physician and cessation educator.

### Measures

The primary outcome of this study was the self-reported 7-day point prevalence abstinence rate at week 24 after enrollment. The secondary outcomes included the medication adherence rate within the cessation course and the proportion of participants who agreed to receive pharmacotherapy in the SDM and non-SDM groups^27^. The medication adherence rate was assessed by whether the participant completed all 16 weeks of medication subsidized or continuous medication usage until abstinence during the 24-week period.

### Statistical analysis

Demographic characteristics were analyzed by conducting independent t-tests for numerical data and chi-squared tests for categorical data. The difference in the proportion of 7-day point prevalence abstinence at week 24, medication adherence rates, and participants who agreed to receive pharmacotherapy between the SDM and non-SDM groups were also analyzed using the chi-squared test. Univariate analyses were conducted by logistic regression to address the association between 7-day point prevalence abstinence at week 24 and medication adherence rates (only for participants that opted to receive medication) with receiving SDM or not. The results of the logistic regression analysis are reported as odds ratio and 95% confidence interval. The significance threshold for all tests was p<0.05, testing was two-sided, and statistical analyses were performed using SPSS software version 13.0 (IBM SPSS statistic 13). The analysis was not pre-registered, and the results should be considered exploratory.

## RESULTS

Of the 1268 participants, 1187 (93.6%) were included in the primary analysis after excluding those who were lost to follow-up (Figure 1). A total of 982 participants (SDM/non-SDM, 473/509) were willing to receive cessation medication, and 46 (SDM/non-SDM, 19/27) were lost to follow-up among these participants. The demographic characteristics of all the participants and smoking-related variables are shown in Table 1. Among all participants, 610 (48%) agreed to enter the SDM model [508 (83.3%) men; mean age (SD), 53.54 (12.48) years], and 509 (83.4% of the SDM group) agreed to receive cessation medication. A total of 658 (52%) refused to enter the SDM model [565 (85.9%) men; mean age (SD), 53.07 (12.61) years], and 473 (71.9% of the non-SDM group) agreed to receive cessation medication. There were no significant differences in age, sex, number of cigarettes per day, or FTND between the two groups.

**Table 1:** Demographic characteristics of adult smokers included in the primary analysis, Taipei, Taiwan, January 2019 to June 2021 (N=1187)

*Characteristics*	*With SDM* *n (%)*	*Without SDM* *n (%)*	*p*
**Sex**			0.213
Female	102 (16.7)	93 (14.1)	
Male	508 (83.3)	565 (85.9)	
**Age** (years), mean (SD)	53.54 (12.48)	53.07 (12.61)	0.369
**Cigarettes/day**, mean (SD)	20.58 (10.63)	21.19 (12.27)	0.482
**FTND^a^,** mean (SD)	5.2 (2.54)	5.21 (2.56)	0.992

SDM: shared decision-making.

a FTND: Fagerström test for nicotine dependence. A six-item scale with four binary items scored 0 or 1, and two multiple-choice items scored from 0 to 3. The score ranges from 0 to 10, with values close to 5 representing a moderate level of cigarette dependence.

**Figure 3:**
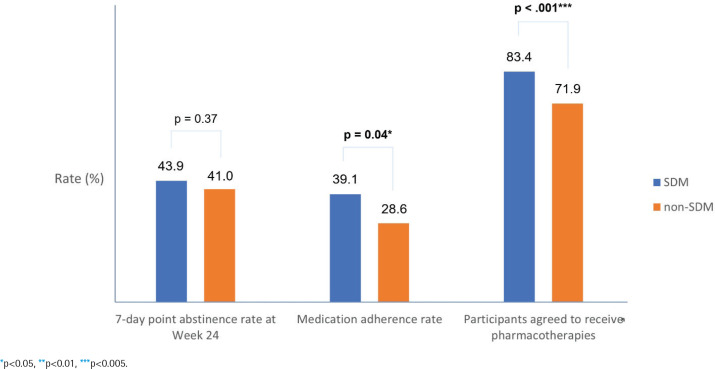
Bar graph of the comparison of 7-day point abstinence rate at week 24, medication adherence rate, and participants agreed to receive pharmacotherapies between the SDM and non-SDM groups

The primary outcome of this study is shown in Figure 3. Among the 936 (SDM/non-SDM, 490/446) participants who were willing to use the cessation medication, 397 [SDM/non-SDM, 201 (41.0%)/196 (43.9%)] were able to reach the 7-day point prevalence abstinence at week 24. The association between the 7-day point prevalence abstinence rate at week 24 and receiving SDM was not significant using logistic regression analysis (OR=0.89; 95% CI: 0.68–1.15; p=0.37).

The secondary outcomes of this study are also shown in Figure 3. Over 70 percent [SDM/non-SDM, 509 (83.4%)/473 (71.9%)] of participants agreed to receive cessation medication, with participants in the SDM group being more likely to agree to use cessation medication. Participants who used the SDM cessation model used cessation medication more frequently (83.4% vs 71.9%, p<0.001). A total of 411 [SDM/non-SDM, 231 (39.1%)/180 (28.6%)] participants completed the cessation medication treatment course and were evaluated to have good medication adherence, while the association of the SDM model with the medication adherence rate was significant (OR=1.32; 95% CI: 1.02–1.71; p=0.04).

## DISCUSSION

The results demonstrated that participants who used the SDM cessation model had a higher willingness to use cessation medication and a higher medication adherence rate than those not using the SDM model. However, the primary outcome of 7-day point abstinence at week 24 was not higher in the SDM group, which was similar to previous studies on substance use disorders (SUD)^28^. These results add valuable knowledge to a poorly researched issue.

Broad endorsement of SDM as high-quality person-centered care has raised the medical society’s awareness^29^. SDM involves patients and clinicians by sharing the best medical evidence, recognizing the values of individuals, and respecting patient autonomy with treatment decisions based on the patient’s best interests. It was supposed to be an effective strategy to increase patient adherence to medication^27^. From the literature review, SDM has been shown to be associated with medication adherence in different diseases. Elwyn et al.^16^ found that SDM between mental health nurses and patients with alcohol use disorders directly enhanced medication adherence. Two other studies also demonstrated higher medication adherence with better SDM in asthma and rheumatological disease^30,31^. On the other hand, lack of SDM was a determinant of negatively affecting direct oral anticoagulant adherence in patients with atrial fibrillation^32^. The SDM cessation model in this study helped participants comply with cessation treatment plans and was the first prospective study revealing that SDM could increase smoking cessation medication adherence, to our knowledge.

Despite the increased medication adherence, the reasons for the insignificant increase in 24-week cessation rate (43.9% to 41%) are interesting for further discussion. First, the cessation rates in the participants of both groups are much higher than those of previous smoking cessation studies, which are about 20%^7^. This finding implies that the participants were patients visiting a cessation clinic in a national medical center with high motivation for smoking cessation. As a result, the high motivation of participants not receiving SDM still contributed to a high cessation rate, which in turn minimized the differences compared to participants receiving SDM. Secondly, only one SDM session in the study was provided, which was during the initial treatment decision. However, health behavior changes such as stopping tobacco smoking need to be observed over a longer period. Joosten et al.^33^ revealed that an SDM intervention composed of five structured sessions helped substance-dependent patients to reach reductions in primary substance use and decrease in addiction severity. With this backdrop, more SDM sessions may be needed in future studies about tobacco use disorder.

Although SDM has been shown to improve outcomes in somatic health conditions such as rheumatologic disease control and blood pressure^34,35^, the issue of whether a similar SDM intervention aimed at having more patient involvement in treatment decisions could help substance use disorder (SUD) is controversial. A recent study found that patients with SUD had poorer outcomes when perceiving more participation in treatment decisions^28^. SUD patients showed a higher likelihood of treatment discontinuation at 12 months and substance use at 6 and 12 months when they perceived more involvement in treatment decisions. The authors concluded that patients might experience an excess of responsibility that could negatively influence the outcome of treatment continuation and substance use. Lower self-esteem and a submissive character were observed in SUD patients^25,36^, and the clinical outcomes might become worse if they perceive more responsibilities than desired in treatment decisions. Tobacco usage and SUD are addiction disorders where patients may have a tendency not to completely abandon consumption, and too much responsibility during SDM might result in more self-deception. The results demonstrated similar outcomes in participants with tobacco use compared to patients with SUD, where more involvement of patients in treatment decisions did not bring the expected better outcome. Patients might want to be involved to some extent in the decision-making process, but not more than they desire. Further research on matching the preferences and perceptions of SDM for smoking cessation patients is warranted.

### Limitations

This cohort study had several limitations. First, the primary outcome relied on self-reported point abstinence and was not biochemically confirmed due to the COVID-19 pandemic and related restrictions among health providers. Second, the cost-effectiveness of SDM should be studied, as bias might be derived from the greater intensity, effort, and time of counseling in the SDM group, although these key features of SDM may be beneficial for increasing the cessation rate. Third, active participation in SDM programs and processes may cause self-selection bias and become a confounding factor in decision-making outcomes. Generalizability was limited considering the representability of cessation service participants to other populations. Lastly, multivariable regression analysis was conducted but the result showed no significance and therefore was not listed in the current study. Possible confounders, including gender, education level, and population, should be studied for the best application and adjustment of the model.

## CONCLUSIONS

Among adults who smoke, SDM was positively associated with higher medication adherence and the proportion of participants who agreed to receive pharmacotherapies. However, the association of SDM with the 7-day point prevalence of abstinence at week 24 was not statistically significant. Longer follow-up studies are needed to understand the association of the SDM intervention with absolute abstinence.

## Data Availability

The data supporting this research are available from the authors on reasonable request.

## References

[1] World Health Organization. WHO global report on trends in prevalence of tobacco use 2000–2025: fourth edition. World Health Organization; 2021. Accessed January 14, 2025. https://iris.who.int/bitstream/handle/10665/348537/9789240039322-eng.pdf?sequence=1

[2] Durazzo TC, Mattsson N, Weiner MW; Alzheimer’s Disease Neuroimaging Initiative. Smoking and increased Alzheimer’s disease risk: a review of potential mechanisms. Alzheimers Dement. 2014;10(suppl 3):S122-S145. doi:10.1016/j.jalz.2014.04.009 24924665 PMC4098701

[3] Patanavanich R, Glantz SA. Smoking is associated with COVID-19 progression: a meta-analysis. Nicotine Tob Res. 2020;22(9):1653-1656. doi:10.1093/ntr/ntaa082 32399563 PMC7239135

[4] Vardavas CI, Nikitara K. COVID-19 and smoking: a systematic review of the evidence. Tob Induc Dis. 2020;18(March):20. doi:10.18332/tid/119324 32206052 PMC7083240

[5] GBD 2019 Diseases and Injuries Collaborators. Global burden of 369 diseases and injuries in 204 countries and territories, 1990-2019: a systematic analysis for the Global Burden of Disease Study 2019. Lancet. 2020;396(10258):1204-1222. doi:10.1016/S0140-6736(20)30925-9 33069326 PMC7567026

[6] Sleeman KE, de Brito M, Etkind S, et al. The escalating global burden of serious health-related suffering: projections to 2060 by world regions, age groups, and health conditions. Lancet Glob Health. 2019;7(7):e883-e892. doi:10.1016/S2214-109X(19)30172-X 31129125 PMC6560023

[7] Rigotti NA, Kruse GR, Livingstone-Banks J, Hartmann-Boyce J. Treatment of tobacco smoking: a review. JAMA. 2022;327(6):566-577. doi:10.1001/jama.2022.0395 35133411

[8] Catz SL, Jack LM, McClure JB, et al. Adherence to varenicline in the COMPASS smoking cessation intervention trial. Nicotine Tob Res. 2011;13(5):361-368. doi:10.1093/ntr/ntr003 21350041 PMC3082504

[9] Patnode CD, Henderson JT, Thompson JH, Senger CA, Fortmann SP, Whitlock EP. Behavioral counseling and pharmacotherapy interventions for tobacco cessation in adults, including pregnant women: a review of reviews for the U.S. preventive services task force. Ann Intern Med. 2015;163(8):608-621. doi:10.7326/M15-0171 26389650

[10] Anthenelli RM, Benowitz NL, West R, et al. Neuropsychiatric safety and efficacy of varenicline, bupropion, and nicotine patch in smokers with and without psychiatric disorders (EAGLES): a double-blind, randomised, placebo-controlled clinical trial. Lancet. 2016;387(10037):2507-2520. doi:10.1016/S0140-6736(16)30272-0 27116918

[11] Baker TB, Piper ME, Smith SS, Bolt DM, Stein JH, Fiore MC. Effects of combined varenicline with nicotine patch and of extended treatment duration on smoking cessation: a randomized clinical trial. JAMA. 2021;326(15):1485-1493. doi:10.1001/jama.2021.15333 34665204 PMC8527361

[12] Cropsey KL, Wolford-Clevenger C, Sisson ML, et al. A pilot study of nicotine replacement therapy sampling and selection to increase medication adherence in low-income smokers. Nicotine Tob Res. 2021;23(9):1575-1583. doi:10.1093/ntr/ntab029 33608735 PMC8372633

[13] Raupach T, Brown J, Herbec A, Brose L, West R. A systematic review of studies assessing the association between adherence to smoking cessation medication and treatment success. Addiction. 2014;109(1):35-43. doi:10.1111/add.12319 23919621

[14] Pacek LR, McClernon FJ, Bosworth HB. Adherence to pharmacological smoking cessation interventions: a literature review and synthesis of correlates and barriers. Nicotine Tob Res. 2018;20(10):1163-1172. doi:10.1093/ntr/ntx210 29059394 PMC6121917

[15] Kim N, McCarthy DE, Loh WY, et al. Predictors of adherence to nicotine replacement therapy: Machine learning evidence that perceived need predicts medication use. Drug Alcohol Depend. 2019;205:107668. doi:10.1016/j.drugalcdep.2019.107668 31707266 PMC6931262

[16] Elwyn G, Durand MA, Song J, et al. A three-talk model for shared decision making: multistage consultation process. BMJ. 2017;359:j4891. doi:10.1136/bmj.j4891 29109079 PMC5683042

[17] Lancaster T, Stead LF. Individual behavioural counselling for smoking cessation. Cochrane Database Syst Rev. 2017;3(3):CD001292. doi:10.1002/14651858.CD001292.pub3 12137623

[18] Lindson N, Thompson TP, Ferrey A, Lambert JD, Aveyard P. Motivational interviewing for smoking cessation. Cochrane Database Syst Rev. 2019;7(7):CD006936. doi:10.1002/14651858.CD006936.pub4 31425622 PMC6699669

[19] Caponnetto P, Maglia M, Floresta D, et al. A randomized controlled trial to compare group motivational interviewing to very brief advice for the effectiveness of a workplace smoking cessation counseling intervention. J Addict Dis. 2020;38(4):465-474. doi:10.1080/10550887.2020.1782564 32634052

[20] Joosten EA, DeFuentes-Merillas L, de Weert GH, Sensky T, van der Staak CP, de Jong CA. Systematic review of the effects of shared decision-making on patient satisfaction, treatment adherence and health status. Psychother Psychosom. 2008;77(4):219-226. doi:10.1159/000126073 18418028

[21] Hollands GJ, Naughton F, Farley A, Lindson N, Aveyard P. Interventions to increase adherence to medications for tobacco dependence. Cochrane Database Syst Rev. 2019;8(8):CD009164. doi:10.1002/14651858.CD009164.pub3 31425618 PMC6699660

[22] Moyo F, Archibald E, Slyer JT. Effectiveness of decision aids for smoking cessation in adults: a quantitative systematic review. JBI Database System Rev Implement Rep. 2018;16(9):1791-1822. doi:10.11124/JBISRIR-2017-003698 30204670

[23] Siewchaisakul P, Luh DL, Chiu SYH, Yen AMF, Chen CD, Chen HH. Smoking cessation advice from healthcare professionals helps those in the contemplation and preparation stage: an application with transtheoretical model underpinning in a community-based program. Tob Induc Dis. 2020;18(July):57. doi:10.18332/tid/123427 32641923 PMC7336865

[24] Aveyard P, Massey L, Parsons A, Manaseki S, Griffin C. The effect of Transtheoretical Model based interventions on smoking cessation. Soc Sci Med. 2009;68(3):397-403. doi:10.1016/j.socscimed.2008.10.036 19038483

[25] Joosten EA, De Jong CA, de Weert-van Oene GH, Sensky T, van der Staak CP. Shared decision-making: increases autonomy in substance-dependent patients. Subst Use Misuse. 2011;46(8):1037-1038. doi:10.3109/10826084.2011.552931 21370962

[26] Chen KL, Hsu YC, Li YH, et al. Shared Decision-Making Model for adolescent smoking cessation: pilot cohort study. Int J Environ Res Public Health. 2021;18(20):10970. doi:10.3390/ijerph182010970 34682716 PMC8536195

[27] Sandman L, Granger BB, Ekman I, Munthe C. Adherence, shared decision-making and patient autonomy. Med Health Care Philos. 2012;15(2):115-127. doi:10.1007/s11019-011-9336-x 21678125

[28] Serrano-Pérez P, Rivero-Santana A, Daigre-Blanco C, et al. Shared decision making in patients with substance use disorders: a one-year follow-up study. Psychiatry Res. 2023;329:115540. doi:10.1016/j.psychres.2023.115540 37857131

[29] Dennison Himmelfarb CR, Beckie TM, Allen LA, et al. Shared decision-making and cardiovascular health: a scientific statement from the American Heart Association. Circulation. 2023;148(11):912-931. doi:10.1161/CIR.0000000000001162 37577791

[30] Kremer IEH, Hiligsmann M, Carlson J, et al. Exploring the cost effectiveness of shared decision making for choosing between disease-modifying drugs for relapsing-remitting multiple sclerosis in the Netherlands: a state transition model. Med Decis Making. 2020;40(8):1003-1019. doi:10.1177/0272989X20961091 33174513 PMC7672783

[31] Topaz M, Zolnoori M, Norful AA, Perrier A, Kostic Z, George M. Speech recognition can help evaluate shared decision making and predict medication adherence in primary care setting. PLoS One. 2022;17(8):e0271884. doi:10.1371/journal.pone.0271884 35925922 PMC9352008

[32] Moudallel S, van den Bemt BJF, Zwikker H, et al. Association of conflicting information from healthcare providers and poor shared decision making with suboptimal adherence in direct oral anticoagulant treatment: a cross-sectional study in patients with atrial fibrillation. Patient Educ Couns. 2021;104(1):155-162. doi:10.1016/j.pec.2020.06.016 32622691

[33] Joosten EA, de Jong CA, de Weert-van Oene GH, Sensky T, van der Staak CP. Shared decision-making reduces drug use and psychiatric severity in substance-dependent patients. Psychother Psychosom. 2009;78(4):245-253. doi:10.1159/000219524 19468259

[34] Saheb Kashaf M, McGill ET, Berger ZD. Shared decision-making and outcomes in type 2 diabetes: a systematic review and meta-analysis. Patient Educ Couns. 2017;100(12):2159-2171. doi:10.1016/j.pec.2017.06.030 28693922

[35] Yun D, Choi J. Person-centered rehabilitation care and outcomes: a systematic literature review. Int J Nurs Stud. 2019;93:74-83. doi:10.1016/j.ijnurstu.2019.02.012 30870614

[36] Alavi HR. The role of self-esteem in tendency towards drugs, theft and prostitution. Addict Health. 2011;3(3-4):119-124. 24494126 PMC3905528

